# A narrative review of the use of PROMs and machine learning to impact value-based clinical decision-making

**DOI:** 10.1186/s12911-025-03083-8

**Published:** 2025-07-04

**Authors:** Michal Pruski, Simone Willis, Kathleen Withers

**Affiliations:** 1 School of Health Sciences, The University of Manchester, Manchester, UK; 2 CEDAR, Cardiff and Vale UHB, Cardiff, UK; 3 Specialist Unit for Review Evidence, Cardiff University, Cardiff, UK; 4 School of Engineering, Cardiff University, Cardiff, UK

**Keywords:** Prudent healthcare, Decision-making, Value in health, Algorithms, Prediction, Patient reported outcomes

## Abstract

**Purpose:**

This review summarises the studies which combined Patient Reported Outcome Measures (PROMs) and Machine Learning statistical computational techniques, to predict patient post-intervention outcomes. The aim of the project was to inform those working in value-based healthcare how Machine Learning can be used with PROMs to inform clinical practice.

**Methods:**

A systematic search strategy was developed and run in six databases. The records were reviewed by a reviewer if they matched the review scope, and these decisions were scrutinised by a second reviewer.

**Results:**

82 records pertaining to 73 studies were identified. The review highlights the breadth of PROMs tools investigated, and the wide variety of Machine Learning techniques utilised across the studies. The findings suggest that there has been some success in predicting post-intervention patient outcomes. Nevertheless, there is no clear best performing Machine Learning approach to analyse this data, and while baseline PROMs scores are often a key predictor of post-intervention scores, this cannot always be assumed to be the case. Moreover, even when studies looked at similar conditions and patient groups, often different Machine Learning techniques performed best in each study.

**Conclusion:**

This review highlights that there is a potential for PROMs and Machine Learning methodology to predict patient post-intervention outcomes, but that best performing models from other previous studies cannot simply be adopted in new clinical contexts.

**Supplementary information:**

The online version contains supplementary material available at 10.1186/s12911-025-03083-8.

## Background

Wales is at the forefront of collecting patient reported outcome measures (PROMs) in clinical practice on a national level [1–4]. As the digital revolution progresses, novel technologies, such as machine learning (ML) and other artificial intelligence (AI) techniques, offer new possibilities of utilising healthcare data. This includes both facilitating big data research using routinely collected data, and the application of these findings to facilitate patient care.

Being a subset of AI, ML is a group of computational techniques which allows researchers to better scrutinise their data. While ML techniques utilise various levels of supervision (labelling the data), they are all potent tools that allow the identification of relationships in the data by creating models. Such models could potentially be used to make predictions for e.g. clinicians to prognosticate how patients might perform under two different treatment regimes. Therefore, one of the potential clinical uses of ML is to identify the best care pathways for individual patients. Consequently, within the context of this work, ML is best understood as a group of complex statistical modelling techniques.

Value-Based healthcare is concerned with the real-life impact of clinical decisions to achieve better outcomes and experiences for service users [5]. As such, it recommends assessing patients’ ability to carry out every-day activities as meaningful measures, over changes in e.g. biochemistry markers. Value-Based healthcare also encourages prudent use of limited resources, and while this is a complex principle, it is clear that interventions need to make an impact to be considered valuable. PROMs, which often focus on measuring patients’ symptom severity and ability to undertake everyday activities, can help to identify such impactful interventions.

In Wales, value-based healthcare application is directed by four principles of Prudent Healthcare [6]:

1. Achieve health and wellbeing with the public, patients and professionals as equal partners through co-production
2. Care for those with the greatest health need first, making the most effective use of all skills and resources
3. Do only what is needed, no more, no less; and do no harm
4. Reduce inappropriate variation using evidence-based practices consistently and transparently

The ability to predict which patients might benefit from a given intervention could help to bring these principles into practice. Having evidence-based predictions will allow patients and clinicians to make better informed decisions. Such predictive models will help identify those of greatest need. They could help ascertain which interventions potentially do not offer any benefit to specific patients. Finally, predictive models might help to usher in precision medicine to help highlight instances of appropriate variation of treatment recommendations.

While some reviews have attempted to address aspects of this topic, they did not utilise comprehensive literature search strategies and so painted only a limited picture of such applications [7, 8]. One of these reviews did specifically look at the ability of ML in combination with PROMs to predict patient outcomes [7]. However, this study only interrogated two databases (PubMed and Scopus) and discussed fifteen studies. The other review focused only on the use of PROMs in clinical AI trials and searched only the ClinicalTrials.gov register [8]. It did not specifically look at the ability of PROMs and ML to predict patient outcomes.

This review was undertaken with the objective to inform stakeholders, such as decision-makers and researchers working in value-based healthcare, about the current applications of ML techniques to PROMs data. It particularly aimed to inform stakeholders how PROMs data collected during routine clinical practice can be utilised in a value-based healthcare system. This information can be used to identify areas of interest for undertaking similar projects, as well as in identifying approaches which have historically not proven to be successful. It is hoped that this review will provide a quick reference guide for those looking to identify studies in their field of interest.

This review looked at published studies which combined PROMs and ML to predict patients’ post-intervention outcomes (Table 1). The review included studies where PROMs were used either as outcome measures and/or as predictors in the ML models. A broad understanding of ‘healthcare intervention’ has been adopted, inclusive of such phenomena as a hospital stay in specialist care, surgery or psychological interventions (Table 1). Nevertheless, due to this already broad scope, the review did not consider other types of outcomes, such as costs or care-giver wellbeing. Moreover, due to the volume of identified studies we did not consider other predictor variables or outcome measures that might have been used in the identified studies.

**Table 1:** Review scope

	Inclusion	Exclusion
**Population**	Patients receiving any healthcare intervention e.g. - Surgical intervention - Psychological intervention - Hospital stay - Pharmacological treatment Patients within any part of the healthcare system (e.g. primary care, secondary care)	
**Phenomenon of interest**	Combining ML with PROMs There are three scenarios where ML and PROMs could be combined: 1. PROMs data collected pre-intervention or phenomenon, followed by ML predicting clinical outcome (i.e. entering PROMs data and using ML for the prediction of clinical outcome) 2. Using ML for the prediction of post-intervention or phenomenon PROMs (ML can be using any kind of data) 3. Pre-intervention or phenomenon PROMs with ML predicting post-intervention PROMs PROMs can be combined with other data (e.g. clinical data, demographic) All validated PROMs ML is defined as statistical computational modelling techniques utilising various levels of user supervision.	PROMs that have not been validated. If only a single item scale such as one visual analogue scale or rating scale was used, e.g. a pain scale was used.
**Outcome**	Patient outcomes - Clinical outcomes - Mortality - PROMs Healthcare system - Prioritisation of patients - Waiting list - Cost savings - Cost effectiveness	Caregiver outcomes Financial outcomes Diagnosis prediction
**Study design**	Any study design (including quantitative, qualitative, mixed-methods, case study, protocols, systematic reviews, rapid reviews; including conference abstracts and registered trials)	Narrative reviews Letters Editorials
**Year**	2000 onwards	Studies published before 2000
**Language**	English language	Not English language

## Main text

### Methods

As the review aimed to inform stakeholders on the topic, we conducted a narrative review. Whilst a scoping review would provide a more rigorous process (e.g. due to a more comprehensive search), we were limited due to time and resources. To increase the methodological rigour of the narrative review, we drew on systematic review methods by systematically searching databases, and having a second reviewer check a proportion of abstracts and data extraction. Due to the narrative nature of the review, it was not registered on PROSPERO.

A search strategy (Supplementary File 1) was developed and run in Medline All (Ovid) to identify relevant records using a combination of free-text and indexed terms. The search included broad terms, such as ‘AI’ to account for the fact that some authors might have used this more general term, rather than specifically describing their techniques as ML. The search was adapted and run in the following five databases: Embase (Ovid), The Cochrane Library, Scopus, IEEE Xplore and ACM Digital Library. The searches were carried out on the 11^th^ October 2023. Records were imported into EndNote 20 and deduplicated [9]. Two reviewers screened studies at title/abstract and full-text using Endnote. One reviewer assessed all records at title/abstract against the inclusion criteria (Table 1). Full texts were obtained, and assessed by one reviewer against the inclusion criteria. At both stages, a second reviewer checked all included records and 10% of excluded records, noting any discrepancies. Discrepancies were resolved through discussion between the two reviewers.

One reviewer extracted key information from each record into a table, which was checked by the second reviewer. Key information was summarised as a narrative and is presented below.

### Results

#### Literature search results

**Fig. 1:**
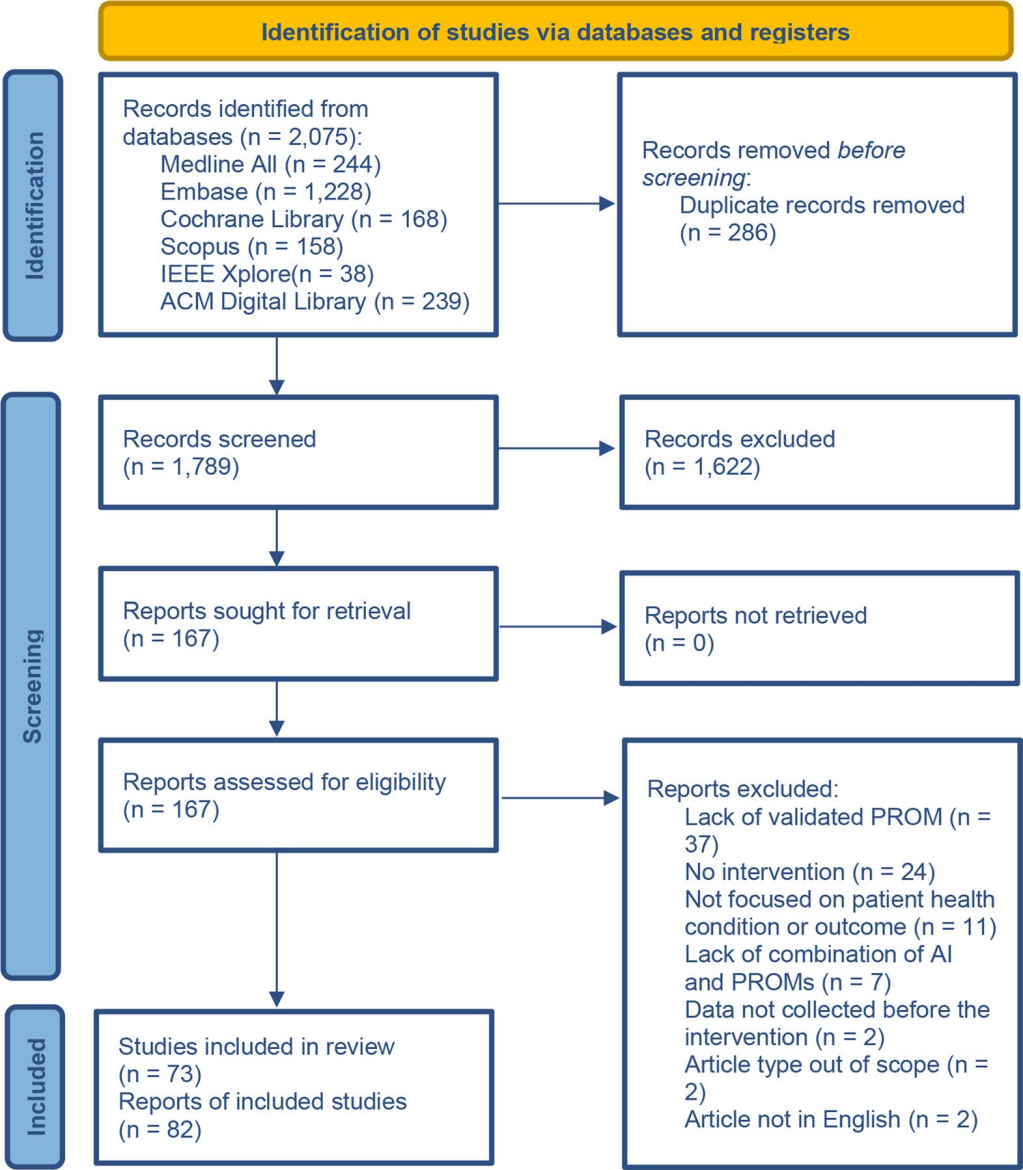
Study selection flow diagram. Adapted from PRISMA. [10, 11]

The searches retrieved a total of 2,075 records, with 1,789 records remaining after deduplication. Following title/abstract screening, 167 records were assessed at full-text, of these, 82 records pertaining to 73 individual studies met the inclusion criteria. The reasons for the exclusion of the remaining 85 records are provided in Fig. 1.

Of the included records, 25 considered the application of ML and PROMs to patients with hip and knee conditions, 14 regarded studies of patients with spinal conditions, and 9 included patients with other musculoskeletal conditions. There were 12 records that looked at cancer patients, four at patients with neurodegenerative conditions, and seven which focused on mental health. The remaining 11 records looked at patients with rheumatoid arthritis, COVID-19, cardiac and respiratory problems, stroke, snake bites, critical care, and the care of the elderly.

#### Findings from the literature

**Fig. 2:**
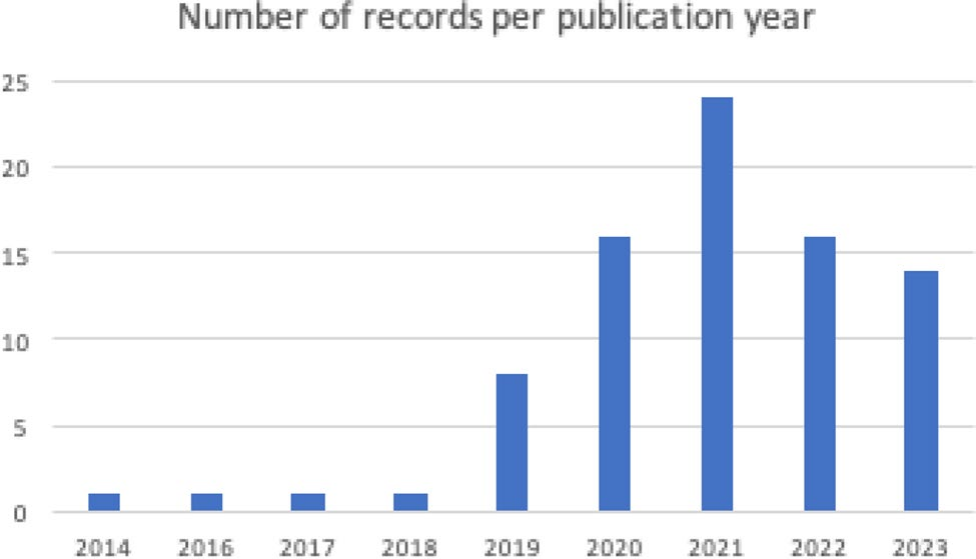
Years of publication for the retrieved records

**Fig. 3:**
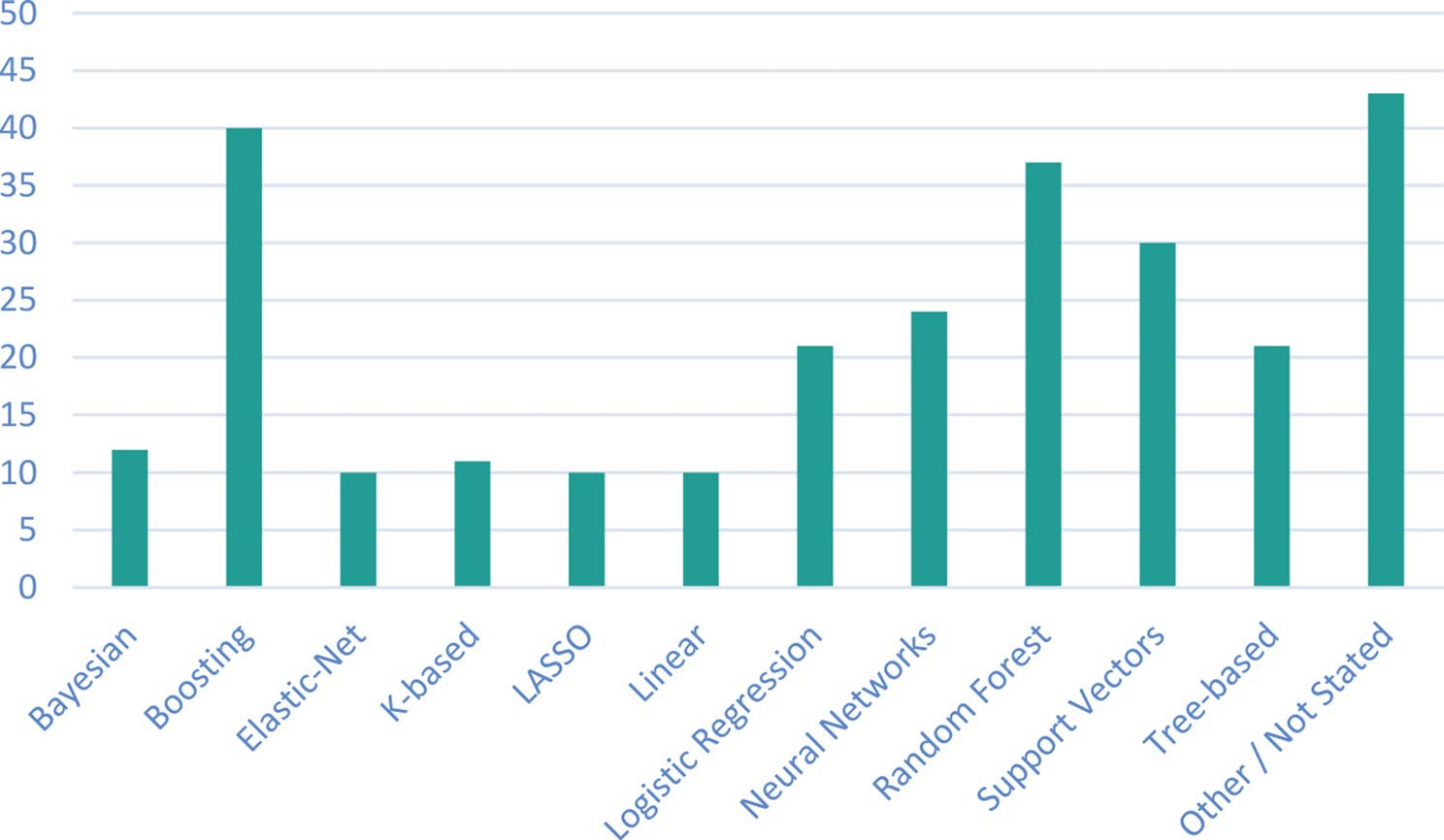
The number of times an ML technique was investigated in the included studies

This section summarises the findings in the identified records, indicating the PROM tools and ML algorithms used. Only the PROM tools, and not specific sub-scores, are reported, and similar ML algorithms are grouped together, to allow for an overall narrative to be presented. These are challenging to summarise due to the heterogeneity in reporting of the studies’ methodologies; for example, some studies described in detail which ML techniques they utilised for feature selection and which for classification when building their ML model, while some studies only stated the generic type of ML algorithm used. Briefly, the majority of records were published between 2019 and 2023 (Fig. 2). Across all studies (Table 2) 220 PROMs were used, of these there were 133 unique PROM tools, with different versions of the same core PROM tool, such as abbreviated versions, counted as different unique PROM tools. Many of the PROM tools were only investigated in single studies, while others, such as Short Form36, were used across a range of studies. Across all studies, 269 ML techniques were mentioned (Table 2). As noted, it is difficult to know how many of these were unique instances due to lack of clarity in the reporting; Fig. 3 provides a summary of the ML techniques used. Of the techniques described in the identified studies, Boosting approaches were most popular, followed by Random Forest and Support Vector approaches, which have been historically ‘three of the most powerful machine learning methods with demonstrated high predictive accuracies in many application domains’ [12]. The following sections provide a brief overview of the included studies, and discussion of the main findings regarding the PROM tools used and the ML techniques employed.

**Table 2:** Summary of PROM tools and ML techniques used in the included studies

Theme	First Author & Year	PROMs considered	ML techniques
Hips & Knees	Bini 2019 [13]	KOOS, HOOS, VR-12	Unsupervised ML for feature selection, K-Means analysis for cluster identification
	Fontana 2019 [14]	**KOOS, HOOS, SF-36**, EQ-5D, WOMAC	Logistic LASSO, RF, Linear SVM
	Harris 2021 [15]	**KOOS**, AUDIT, PHQ-2, EQ-5D-5 L	LR, LASSO, GBoost, QDA
	Heisinger 2020 [16]	KOOS, WOMAC	NN
	Jayakumar 2020 & 2021 [17, 18]	PROMIS, KOOS, PHQ −2 and -9, GAD −2 and −7, **Knee Osteoarthritis Decision Quality Instrument**	Not stated
	Jiang 2021 [19]	**WOMAC, SF-36**	Penalised Regression, Kernel Ridge Regression, RF, Reinforcement Learning Trees, List-Based Dynamic Treatment Regime, Residual Weighted Learning, Bayesian Additive Regression Trees, Zero-Order models
	Kastrup 2023 (protocol) & NCT04332055 (registry entry) [20, 21]	**OKS**, **OHS**, EQ-5D-3 L and the Shared Decision Making Questionnaires Questionniare-9	Not stated
	Kunze 2021 [22]	HOS, mHHS	Stochastic GBoost, RF, SVM, NN, ENPLR
	Kunze 2021 [23]	**HOS**, mHHS	StochasticGBoost, RF, SVM, NN, ENPLR
	Kunze 2021 [24]	**HOS**, mHHS, iHOT	Stochastic GBoost, RF, SVM, Adaptive GBoost, NN, ENPLR
	Kunze 2022 [25]	HOS, mHHS, **iHOT**	Stochastic GBoost, RF, SVM, XGBoost, NN, ENPLR
	Lopez 2021 (systematic review) [26]	HOOS, KOOS, SF-36	NN, Regression, Cluster Analysis, SVM, DT, Boosting, Bayesian Networks
	Milella 2022 [27]	**SF-12**	XGBoost, DT
	Ramkumar 2020 & 2021 [28, 29]	**HOS, mHHS, iHOT**	RF
	Ramkumar 2021 & Karnuta 2021 [30, 31]	**IKDC**, **KOS-ADL**, **SF-36**	LR, RF, GNB, XGBoost, Sigmoid XGBoost, Isotinic XGBoost, Top Three Ensemble methodology
	Ribbons 2023 (protocol) [32]	**WOMAC**, Depression Anxiety and Stress Scales-21, Pain Catastrophizing Scale, Brief Resilience Scale, Committed Action Questionnaire-8, Valued Living Scale, **SF-12 version 2**, Medical Outcome Study Social Support Survey, University of California Los Angeles Activity Scale, AUDIT	linear predictive models, DT, RF, GBoost, NN, Bayesian Soft Decision Trees
	Sergooris 2023 (protocol) [33]	**HOOS, SF-36, Global Perceived Effect Scale, Patient Specific Functioning Scale, ** FACS-D, TSK-17, IEQ, Childhood Trauma Questionnaire, Mini International Neuropsychiatric Interview Simplified, Hospital Anxiety and Depression Scale, General Self-Efficacy Scale, Perceived Stress Scale	LASSO, DT, GBoost, recurrent NN
	Sniderman 2021 [34]	**HOOS**	LASSO
	Zhang 2022 [35]	WOMAC, SF-36	RF, XGBoost, SVM, LR, LASSO
	Zhou 2022 (protocol) & 2022 [36, 37]	**VR-12**, EQ-5D-3 L	LR, Classification Tree, XGBoost tree, RF
Spinal Conditions	Ames 2019 [38]	**ODI, Scoliosis Research Society-22, Optum SF-36v2 Health Survey**	Hierarchical clustering analysis
	Chan 2021 & 2022 [39, 40]	ODI, EQ-5D, North American Spine Society Satisfaction Questionnaire	K-Means Clustering
	Durand 2020 [41]	Scoliosis Research Society-22, ODI, SF-36	RF, ENR, LR, SVM with radial kernels, SVM with linear kernels
	Janssen 2021 [42]	SF-36, HADS, ODI, PCS	RF
	Liew 2020 [43]	**NDI**, **EQ-5D**, MSPQ, SES	Stepwise Regression, LASSO, Boosting, MuARS
	Merali 2019 [44]	**mJOA**, SF-36, **SF-6D**, NDI	RF, SVM and LR, Simple DT and NN; RF used for feature selection
	Muller 2021 [45]	COMI	LASSO cross validation, Ridge cross validation
	Rogers 2019 [46]	mJOA	RF, SVM, NN, DT
	Siccoli 2019 [47]	ODI	RF, XGBoost, Bayesian Generalised Linear Models, simple Generalised Linear Models, Boosted Trees, KNN, NN with a single hidden layer
	Sundararajan 2019 [48]	ODI	ENR
	Yagi 2022 [49]	**JOABPEQ**	Generalised Linear Regression, GLMM, LR, linear SVM, single-layer NN, Random Tree, ‘linear-AS’, ‘tree-AS’, XGBoost Linear, XGBoostTree, Chi-Squared Automatic Interaction Detection, classification trees, regression trees
	Zhang 2021 [50]	**mJOA**	DT, RF, ET, Adaboost, GBoost DT, Bernoulli Naïve Bayes, GNB, Passive Aggressive, QDA, Linear Discriminant Analysis, Linear SVM, SVM, KNN, SGD; feature processors: select percentile, select rate, Linear SVM pre-processor, ET pre-processor, Fast Independent Component Analysis, Feature Agglomeration, Principal Component Analysis
Other Musculoskeletal	Allaart 2023 (protocol) [51]	Constant-Murley Score, ASES, UCLA, OSS, WORC, DASH	Bayes Point Machine, Boosted DT, Penalised LR, NN, SVM
	Dipnall 2021 (protocol) [52]	WHODAS, EQ-5D-5 L	Linear Mixed Models, Generalised Linear Mixed Models, Longitudinal Multi-Level Factorization Machines Model, Longitudinal Support Vector Regression, Mixed Effects RF
	Kong 2020 [53]	**RMDQ**	LASSO
	Kumar 2020 [54]	SPADI, **ASES**, Simple Shoulder Test, **Constant-Murley Score**, **UCLA**	LR, XGBoost, Wide and Deep ML techniques
	Loos 2022 [55]	**MHQ**	LR, RF, GBoost
	Lu 2023 [56]	ASES, Single Assessment Numeric Evaluation, Constant-MurleyScore	RF
	Polce 2021 [57]	Single AssessmentNumeric Evaluation	Stochastic GBoost, RF, SVM, NN, ENPLR
	Verma 2021 [58]	**RMDQ**, **Work-ability index**, **PSEQ**, **FABQ**, **Global Perceived Effect Scale, **EQ-5D	XGBoost
	Verma 2022 [59]	HADS, Multidimensional Pain Inventory, Pain Disability Index, Psychological Inflexibility in Pain Scale, SF-36	For regression: LR, Passive Aggressive Regression, RF Regression, Stochastic Gradient Descent Regression, AdaBoost, Support Vector Regression, XGBoost Regression; for classification: balanced RF and Random Under-sampling Boosting classifiers, both with DT as base estimators
	Vo 2023 (protocol) [60]	PROMIS, painDETECT, ODI, StarT Back Tool, Fear Avoidance Beliefs Questionnaire, Chronic Pain Acceptance Questionnaire, PCS, Interoception: Multidimensional Assessment of Interoceptive Awareness, PHQ, GAD-2, Perceived Stress Scale, Primary Care Post-traumatic Stress Disorder Screen, HEAL Positive Outlook, Global Physical Activity Questionnaire	NN, SVM
Cancer	Cunha 2021 [61]	Edmonton Symptom Assessment System, EORTC QLQ-C30	Feature selection: RF, XGBoost, Analysis of Variance F-Score, Recursive Feature Elimination with Cross-Validation fitted with a SVM, L1penalised Cox; classification: RF, KNN, XGBoost, LR, Voting Classifier (Ensemble)
	DeWees 2020 [62]	**CTCAE**	NN
	Golafshar 2020 [63]	CTCAE	NN
	Iivanainen 2020 & 2020 [64, 65]	CTCAE	XGBoost
	Nuutinen 2023 [66]	**EORTC QLQ-C30**, National Comprehensive Cancer Network distress thermometer	Variable selection: LR; classification: RF
	Qi 2017 [67]	**Expanded Prostate Cancer Index Composite – Short Form tool**	Deep NN
	Rossi 2021 [68]	MDASI	LR
	Savić 2021 [69]	**Prostate Symptom Score, International Index of Erectile Function − 5 item, Life Satisfaction Questionnaire − 11**	Naive Bayes, KNN, SVM, DT, RF; compared centralised and federated models
	Xu 2023 & Pfob 2023 & 2021 [70–72]	**BREAST-Q**	LR, XGBoost, NN (all noted as similarly well performing)
Neurodegenerative	Bougea 2023 [73]	PDQ-39	multivariate linear regression, Autoregressive Integrated Moving Average, Seasonal Autoregressive Integrated Moving Average, Long Short-Term Memory- recurrent NN
	Branco 2022 [74]	SF-36, Treatment Satisfaction Questionnaire for Medication, Fatigue Severity Scale, Beck Depression Inventory-II, Patient-Reported Indices in Multiple Sclerosis	LR, Linear Support Vector, GNB, RF
	Coratti 2023 (protocol) [75]	Spinal Muscular Atrophy Health Index	Not stated
	Rouleau 2020 [76]	**PDQ-39**	SVM
Mental Health	Bremer 2018 [77]	**QIDS**, **PHQ-9**, **EQ-5D**	Feature selection: LASSO; prediction: Linear Regression, Support Vector Regression, regression trees, ridge regression
	Camp 2022 [78]	QOLIE-10, **PHQ-9**	Multilayer Perceptron, RF, SVM, LR with Stochastic Gradient Descent, KNN, GBoost
	Chekroud 2016 [79]	**QIDS**, PHQ-9, EQ-5D	elastic net regularisation with GBoost
	Hufner 2022 [80]	**PHQ-4**	RF, Poisson Regression, KNN
	Kay 2021 [81]	QIDS	RF, GBoost, LR, Deep Learning, J48, Adaboost
	Manikis 2023 [82]	**HADS**, **EORTC QLQ-C30**, Positive and Negative Affect Schedule, Life Orientation Test, Mental Adjustment to Cancer scale, Sense of Coherence Scale, Connor-Davidson Resilience Scale, Mindful Attention Awareness Scale, Quality of Life Questionnaire – Breast Cancer Module, CancerBehaviorInventory – Brief and Post-Traumatic Growth Inventory, Fear of Cancer Recurrence Inventory, Revised Life Orientation Test, Cognitive Emotion Regulation Questionnaire - short, modified Medical Outcomes Study Social Support Survey	Balanced RF
	Martin 2019 [83]	Dimensional Anhedonia Rating Scale, Snaith Hamilton Pleasure Scale	Not stated
Other: Bariatric Surgery	Cao 2020 [84]	SF-36, obesity-related problems scale	Gaussian Bayesian Networks, multinomial discrete Bayesian Networks multivariable LR, Convolution NN
Other: Rheumatoid Arthritis	Curtis 2022 [85]	PROMIS, SF-36	RF, Elastic-Net Regularised Linear Model, XGBoost, SVM, DT
Other: Critical Care	Dias 2014 [86]	**EQ-5D**	Bayesian Networks
Other: Rheumatoid Arthritis	Duong 2022 [87]	HAQ	LASSO, RF
Other: Respiratory	Finnegan 2023 [88]	**Dyspnoea-12**, Centre for Epidemiologic Studies Depression Scale, Trait Anxiety Inventory, Fatigue Severity Scale, St George’s Respiratory Questionnaire, Breathlessness Catastrophizing Scale and Vigilance Scale	Feature selection: elastic net procedure with ranked coefficients; classification: Support Vector Classifier with radial kernel
Other: Cardiac	Frodi 2021 (protocol) [89]	EQ-5D-5 L, Kansas City Cardiomyopathy Questionnaire	RF, K-Neighbors Classifier, Gradient Boosting Classifier, AdaBoost Classifier, Support Vector Classifier, Long Short-Term Memory NN
Other: COVID-19	Gentilotti 2023 [86]	**SF-36**	Principal Component Analysis, LR
Other: Snake Bites	Gerardo 2020 [90]	**Patient-Specific Functional Scale**	Bayesian Belief Network
Other: Stroke	Liao 2022 [91]	**SIS**, Motor Activity Log, Nottingham Extended Activities of Daily Living	RF, KNN, NN, SVM, LR
Other: Care of the Elderly	Stuckenschneider 2022 (protocol) [92]	short falls efficacy scale, Longitudinal Urban Cohort Ageing Study Functional Ability Index, Physical Activity Scale for the Elderly, Life Space Questionnaire, Depression in Old Age Scale, EQ-5D-3 L	Not stated
Other: Stroke	Thakkar 2020 [93]	SIS, Motor Activity Log	KNN, NN

**Bold** - main PROMs outcomes of interest; Underscore - significant outcome predictors or best performing ML techniques; Adaptive Boosting (Adaboost), American Shoulder and Elbow Surgeons score (ASES), Alcohol Use Disorders Identification Test (AUDIT), Core Outcome Measures lndex (COMI), Common Terminology Criteria for Adverse Events (CTCAE), Disabilities of Arm, Shoulder and Hand score (DASH), Decision Trees (DT), Elastic-Net Penalised Logistic Regression (ENPLR), Elastic Net Regression (ENR), European Organisation for the Research and Treatment of Cancer Quality of Life Questionnaire– Core Questionnaire (EORTC QLQ-C30), EuroQoL 5-Dimension (EQ-5D), Fear Avoidance Belief Questionnaire (FABQ), Fear-Avoidance Components Scale (FACS-D), Generalised Anxiety Disorder (GAD), Gradient Boosting (GBoost), Generalised Linear Mixed Model (GLMM), Gaussian Naïve Bayes (GNB), Hospital Anxiety and Depression Scale (HADS), Health Assessment Questionnaire (HAQ), Hip Disability and Osteoarthritis Outcome Score (HOOS), Hip Outcome Score (HOS), Injustice Experience Questionnaire (IEQ), Hip Outcome Tool (iHOT), International Knee Documentation Committee (IKDC) subjective form, Japanese Orthopedic Association Back Pain Evaluation Questionnaire (JOABPEQ), K-Nearest Neighbor (KNN), Knee Injury and Osteoarthritis Outcome Score (KOOS), Knee Outcome Survey–Activities of Daily Living (KOS-ADL), Least Absolute Shrinkage and Selection Operator regression (LASSO), Logistic Regression (LR), MD Anderson Symptom Inventory (MDASI), Modified Harris Hip Score (mHHS), Michigan Hand outcomes Questionnaire (MHQ), modified Japanese Orthopedic Association scale (mJOA), Modified Somatic Perception Questionnaire (MSPQ), Multivariate Adaptive Regression Splines (MuARS), Neck Disability Index (NDI), Neural Networks (NN), Oswestry Disability Index (ODI), Oxford Hip Score (OHS), Oxford Knee Score (OKS), Oxford Shoulder Score (OSS), Pain Catastrophizing Scale (PCS), Patient Health Questionnaire (PHQ), Parkinson’s Disease Questionnaire 39 (PDQ-39), Patient-Reported Outcomes Measurement Information System (PROMIS), Pain Self Efficacy Questionnaire (PSEQ), Quadratic Discriminant Analysis (QDA), Quick Inventory of Depressive Symptomatology (QIDS), Quality of Life in Epilepsy Inventory-10 (QOLIE-10), Random Forest (RF), Roland Morris Disability Questionnaire (RMDQ), Self Efficacy Scale (SES), Short Form (SF), Stochastic Gradient Descent (SGD), Stroke Impact Scale (SIS), Shoulder Pain and Disability Index (SPADI), Support Vector Machine (SVM), Tampa Scale for Kinesiophobia (TSK-17), University of California at Los Angeles shoulder score (UCLA), Veterans RAND 12-item Health Survey (VR-12), WHO Disability Assessment Schedule (WHODAS), Western Ontario and McMaster University Osteoarthritis Index (WOMAC), Western Ontario Rotator Cuff index (WORC), Extreme Gradient Boosting (XGBoost)

#### Hips and knees

There were 25 records which described 20 studies relating to hip and knee conditions, out of which one was a systematic review and for three of these studies only protocols or registry entries were retrieved. There was one record of a systematic review looking at ML powered decision support systems for total hip and knee arthroplasty [26]. It listed twelve studies that considered PROMs as their prediction outputs out of a total 49 studies included in that systematic review. Of these, ten studies were not identified by the systematic search carried out as part of the present review, with the reasons for this discussed later on. Two records pertain to a study looking at the impact of the utilisation of a decision support tool in patients with knee osteoarthritis [17, 18]. One record described a study looking at using wearable sensor data to predict six-week postoperative outcomes in joint replacement patients and only utilised wearable sensor data as predictors in their models [13]. One record described a study that looked at both hip and knee total arthroplasty patients and the authors found all models to perform better than simple heuristics (rules of thumb) [14]. One record described a study that looked at predicting knee replacement surgery from symptomatic and radiographic data [16]. One record related to a study looking at pain and function outcomes 1-year after surgery [15]. One record described a study looking to predict 3-month postoperative outcomes [34]. Two records described a study looking at the capability of radiographic indices in predicting PROM scores [28, 29]. One record looked at the functional improvement in athletes with femoroacetubular impingement syndrome using a two year horizon [24]. Another study also looked at femoroacetubular impingement syndrome patient outcomes [25]. One study considered a visual analogue score for satisfaction as its outcome measure, and did not find PROMs to be important outcome predictors [22]. Conversely, another study found the baseline score of a PROM tool to be an important predictor measure of its outcome value [23]. Two records described a study looking at 1- and 2-year post-osteochondral allograft outcomes in knee cartilage defect patients [30, 31]. This study found diffident models to be the best performing for predicting different outcome PROM scores. Two records related to a study looking at patient willingness to undergo total knee arthroplasty when they had access to a prognostic tool, and utilised a 12-month follow-up window [36, 37]. One study looked to develop a precision medicine approach to managing knee osteoarthritis patients that are either overweight or obese and found different ML algorithms to be best at predicting different outcome measures they had considered [19]. Another study looked at predicting meaningful improvement after total knee arthroplasty [35]. One record described as study that looked to improve treatment decisions in hip and knee surgery patients [27]. Three of the records were of protocols, and for one of these a trial registry entry was also retrieved. One was a protocol looking at developing a decision support tool for patients undergoing total knee arthroplasty and adopting a 3-month postoperative horizon [32]. The second was a protocol of a study looking at osteoarthritis patients undergoing hip arthroplasty, focusing on the impact of traumatic experiences and mental conditions on postoperative outcomes [33]. The remaining two records related to a study looking at osteoarthritis patients with hip or knee problems [20, 21].

**Fig. 4:**
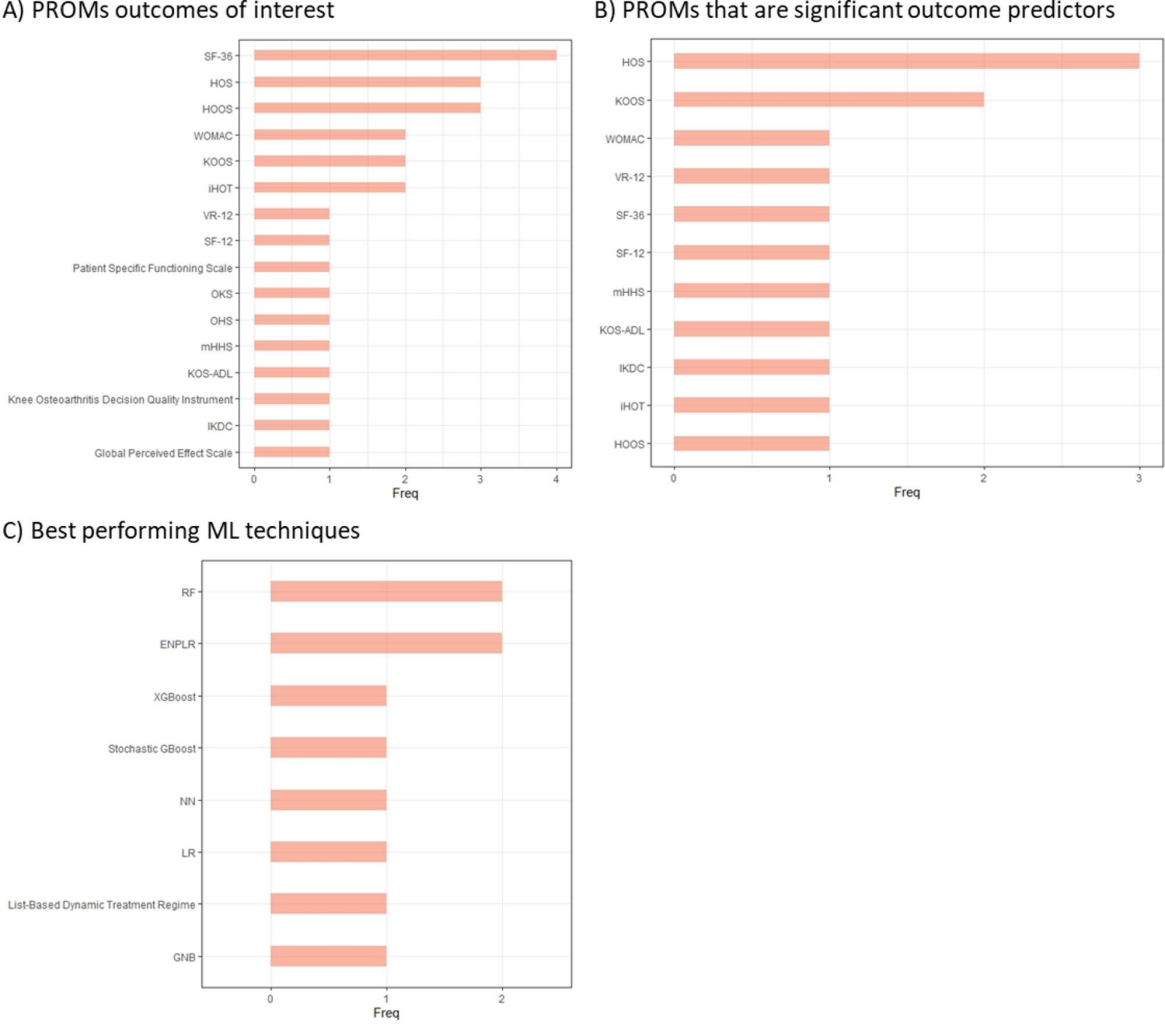
Key PROMs and ML techniques in studies of hip and knee patients. (**A**) PROMs that studies identified as outcomes of interest (**B**) PROMs that studies identified as significant outcome predictors (**C**) ML techniques that studies highlighted as best performing when more than one ML technique was investigated. Elastic-Net Penalised Logistic Regression (ENPLR), Gaussian Naïve Bayes (GNB), Hip Disability and Osteoarthritis Outcome Score (HOOS), Hip Outcome Score (HOS), Hip Outcome Tool (iHOT), International Knee Documentation Committee (IKDC), Knee Injury and Osteoarthritis Outcome Score (KOOS), Knee Outcome Survey–Activities of Daily Living (KOS-ADL), Logistic Regression (LR), Modified Harris Hip Score (mHHS), Neural Networks (NN), Oxford Hip Score (OHS), Oxford Knee Score (OKS), Random Forest (RF), Short Form (SF), Veterans RAND 12-item Health Survey (VR-12), Western Ontario and McMaster University Osteoarthritis Index (WOMAC), Extreme Gradient Boosting (XGBoost)

These studies utilised 42 unique PROM tools, with the most frequently utilised being the Short Form-36 and Knee Injury and Osteoarthritis Outcome Score (both used six times), followed by Hip Disability and Osteoarthritis Outcome Score, Hip Outcome Score, and Western Ontario and McMaster University Osteoarthritis Index, with each used five times (Table 2). Boosting, Random Forests and Neural Networks were the three most often explored algorithms (Table 2). Short Form-36 was the most studied PROMs outcome of interest, while the Hip Outcome Score was the most frequently identified PROMs tool that was a significant predictor of the studied outcomes (Fig. 4). Random Forests and Elastic-Net Penalised Logistic Regression were the most successful ML techniques studied (Fig. 4). Even though the studies pertain to similar conditions their findings are very heterogeneous. For example, four studies that were undertaken by Kunze and colleagues utilised similar PROM tools and tested a similar selection of ML algorithms, but different algorithms were found to perform best in these studies [22–25]. Moreover, different models might perform best for predicting the minimal clinically important difference (the smallest improvement which would be considered worthwhile), patient acceptable syndrome state achievement (achieving a PROM outcome which patients deem acceptable), and substantial clinical benefit (achieving a PROM score change which patients deem significant), and sometimes these ML models do not perform better than simple PROM thresholds [25, 35]. Lastly, in one study which assumed a 4-year pre-operative horizon, the authors found that PROMs only indicated a significant worsening one year before surgery, while radiographic data provided earlier indications of deterioration [16]. However, another study did not find radiographic data to be able to predict PROMs outcomes [28, 29].

#### Spinal conditions

There were 13 records relating to 12 studies which fell into the spinal conditions category. Two studies looked at predicting patient outcomes one year after lumbar spinal stenosis surgery [47, 48]. One record described a study that used radiomics to predict patients’ post-operative outcomes, and used fourteen ML classifiers and seven feature processors [50]. A second reported a study looking at predicting 3-months post-surgery quality of life [46]. A third record looked at 6-,12- and 24-month post-surgical outcomes [44]. Two records, related to a study looking at the outcomes of lumbar spondylitis patients [39, 40]. Two records were of studies looking at adults with spinal deformity undergoing surgery. One study adopted a 2-year outcome horizon, and did not report on any notable outcome predictors [38]. The other study used a 1-year outcome horizon to predict operative vs non-operative management [41]. One record looked at 1- and 2-year postoperative outcomes after lumbar spinal fusion [42]. One record looked at cervical radiculopathy patients [43]. One record looked at the outcomes of patients who underwent decompression surgery for lumbar spinal canal stenosis [49]. One record looked at predicting post-surgical outcomes in patients with degenerative spinal disorders [45].

**Fig. 5:**
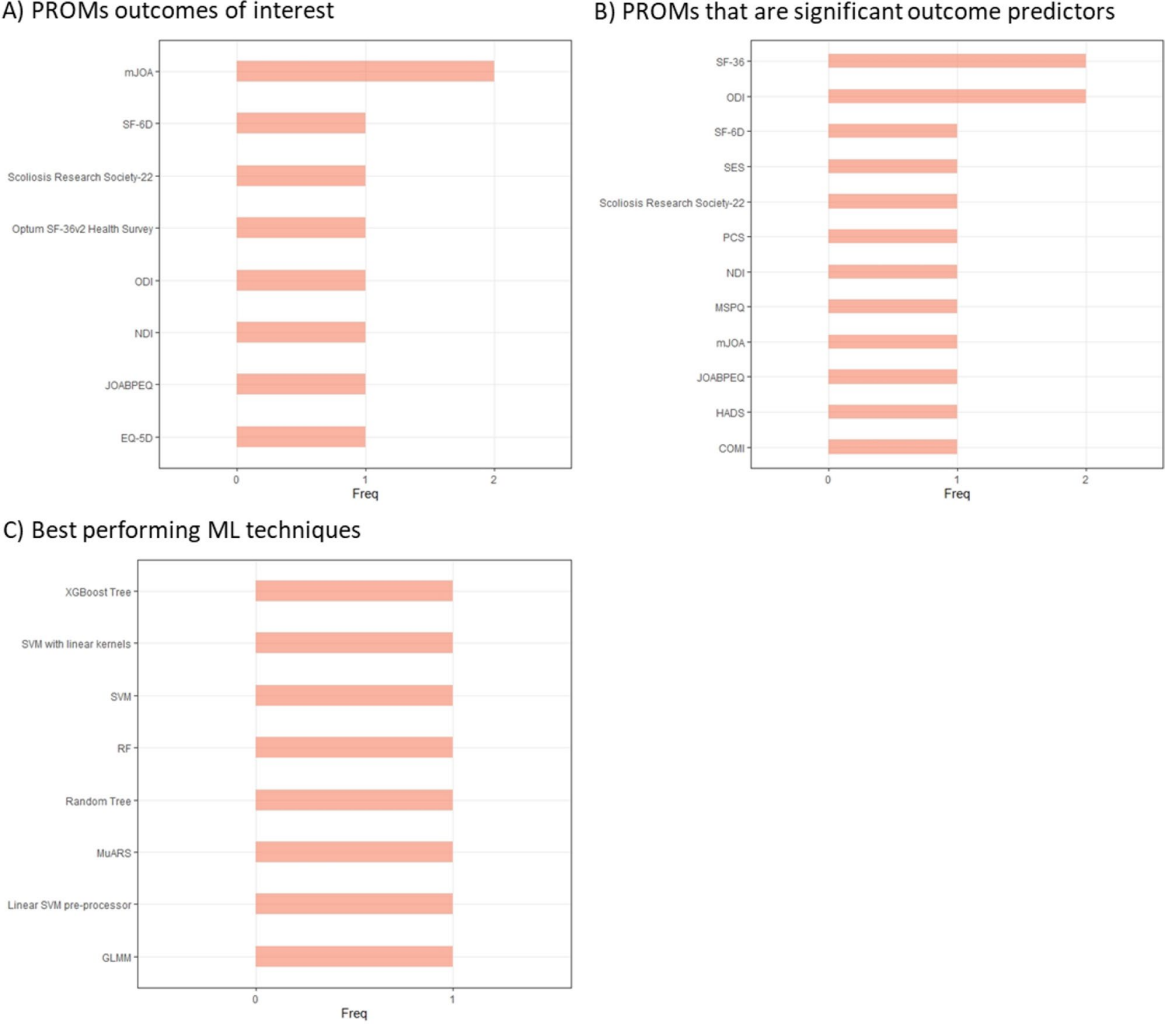
Key PROMs and ML techniques in studies of spinal condition patients. (**A**) PROMs that studies identified as outcomes of interest (**B**) PROMs which studies identified as significant outcome predictors (**C**) ML techniques that studies highlighted as best performing when more than one ML technique was investigated. Core Outcome Measures lndex (COMI), EuroQoL 5-Dimension (EQ-5D), Generalised Linear Mixed Model (GLMM), Hospital Anxiety and Depression Scale (HADS), Japanese Orthopedic Association Back Pain Evaluation Questionnaire (JOABPEQ), modified Japanese Orthopedic Association scale (mJOA), Modified Somatic Perception Questionnaire (MSPQ), Multivariate Adaptive Regression Splines (MuARS), Neck Disability Index (NDI), Oswestry Disability Index (ODI), Pain Catastrophizing Scale (PCS), Random Forest (RF), Self Efficacy Scale (SES), Short Form (SF), Support Vector Machine (SVM), Extreme Gradient Boosting (XGBoost)

There were 15 different PROM tools that were investigated in these studies. The most frequently studied PROM tool was the Oswestry Disability Index (six times), followed by the Short Form-36 and modified Japanese Orthopaedic Association scale, both used three times (Table 2). Boosting, Random Forest, and Support Vector Machines were the most often tested ML approaches (Table 2). The modified Japanese Orthopedic Association scale was the most studied PROM outcome of interest, while the Short Form-36 and Oswestry Disability Index were the most frequently identified PROM tools that were significant predictors of the studied outcomes (Fig. 5). All of the ML techniques which were highlighted as being best performing were only mentioned once in the identified studies (Fig. 5). One study reported that various models had similar performance, so the authors highlighted the most parsimonious of these models [43]. For context, parsimonious models rely on a smaller number of variables, and as such are computationally more efficient and require less data collection. In another case, the authors created an ensemble of the five best models [49]. One study identified two phenotypes of patients: those of high and intermediate disease burden. It found that those with high disease burden demonstrated a greater 24-month horizon improvement on many measures compared to intermediate burden patients, though the higher burden patients had lower satisfaction [39, 40]. Another study found that Modified Somatic Perception Questionnaire and Self Efficacy Scale scores were important predictors of EuroQoL 5-Dimension (EQ-5D) scores, but the baseline EQ-5D score was not [43]. One study failed to make any reliable predictions [46].

#### Other musculoskeletal conditions

There were 10 records relating to 10 studies which described other musculoskeletal conditions. Out of these, three were protocols. Three studies looked at back pain patients. One record was of a study looking at predicting patients’ response to acupuncture [53]. One looked at several outcome measures, but it contained some uncertainty regarding important predictors due to unexplained acronyms [58]. The last study, looked at a range of PROMs and considered seven regression algorithms and two classification algorithms [59]. Three studies looked at patients with shoulder problems. One looked at identifying subgroups of patients undergoing arthroscopic rotator cuff repair [56]. The other two studies looked at outcomes after shoulder arthroplasty. One of these looked at outcomes 2 years post-intervention [57]. The other study considered a range of time points (1 year, 2–3 years, 3–5 years, and more than 5 years post-intervention) for its outcomes [54]. One study looked at predicting outcomes after surgery for thumb carpometacarpal osteoarthritis [55]. One protocol described a study looking at predicting rotator cuff surgery outcomes [51]. The second protocol record described a study looking at predicting fracture outcomes [52]. The last was a protocol of a study to identify phenotypes of lower back pain [60].

As this is a heterogonous collection of studies, information on the most frequently used PROM tools and ML approaches is not provided. The one notable finding is that one study found the pre-intervention American Shoulder and Elbow Surgeons and Shoulder Pain and Disability Index scores, but not the Constant-Murley Score, to be predictive of the post-intervention Constant-Murley Score [54].

#### Cancer

There were 12 records which pertained to nine cancer studies. There were three records which related to two studies including cancer patients in general. One study looked at post-surgery complications in patients suffering from gastrointestinal and lung cancer [68]. Two records related to a study looking at immune-related adverse events in cancer patients receiving immune checkpoint inhibitor therapies [64, 65]. Five records looked specifically at patients suffering from breast cancer. Three of these records were published by members of the same group, utilising patient data from the Mastectomy Reconstruction Outcomes Consortium and looking at one and two year outcomes after breast surgery [70–72]. One record described a study that considered whether using a ML tool improved clinicians’ ability to predict breast cancer patients’ post-treatment quality of life [66]. One study looked at predicting adverse events in breast cancer patients [62]. Three records related to studies concerning prostate cancer patients. Out of these three studies, one study also looked at breast cancer patients, but did not report data relevant to this review with respect to the breast cancer patients [69]. One record looked at predicting adverse events [63]. Another record looked at outcomes in prostate cancer patients receiving stereotactic body radiation therapy [67]. One record, looked at predicting survival in patients suffering from metastatic non-small cell lung cancer [61].

**Fig. 6:**
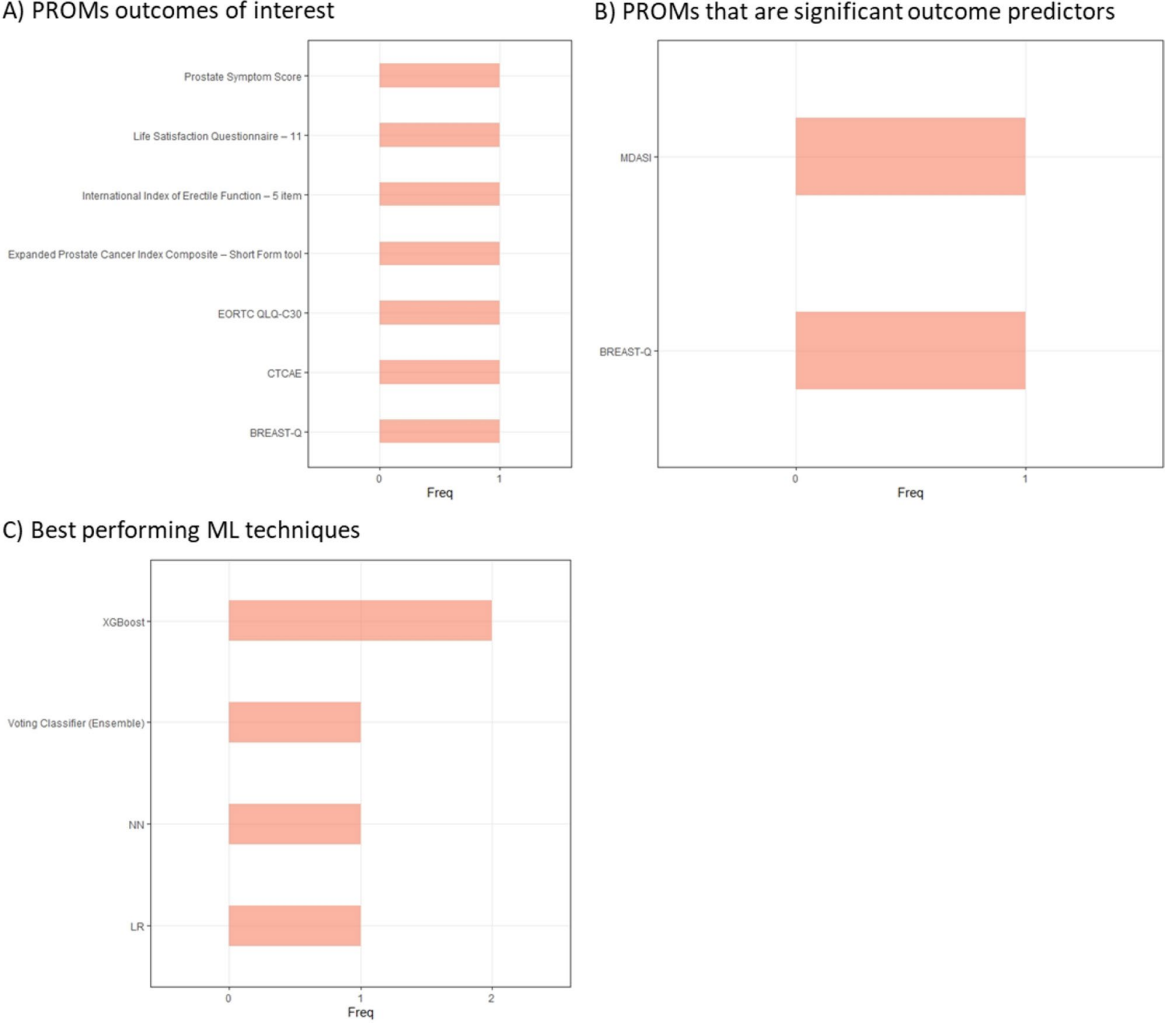
Key PROMs and ML techniques in studies of cancer patients. (**A**) PROMs that studies identified as outcomes of interest (**B**) PROMs which studies identified as significant outcome predictors (**C**) ML techniques that studies highlighted as best performing when more than one ML technique was investigated. Common Terminology Criteria for Adverse Events (CTCAE), European Organisation for the Research and Treatment of Cancer Quality of Life Questionnaire– Core Questionnaire (EORTC QLQ-C30), Logistic Regression (LR), Neural Networks (NN), MD Anderson Symptom Inventory (MDASI), Extreme Gradient Boosting (XGBoost).

Ten unique PROM tools were utilised in these studies, of these Common Terminology Criteria for Adverse Events was utilised three times and the European Organisation for the Research and Treatment of Cancer Quality of Life Questionnaire– Core Questionnaire twice; all other tools were only utilised once (Table 2). Boosting, Logistic Regression, Neural Networks and Random Forests were the most often investigated techniques, each utilised in four studies (Table 2). None of the PROMs which were used as predictors of outcome measures featured more prominently than others, while XGBoost was the most frequently mentioned best performing ML technique (Fig.6). One study, uniquely out of all the studies included in this review, compared centralised and federated models [69]. It found that centralised and federated models performed similarly in predicting short-term quality of life, but that centralised models performed better in making long-term predictions. Authors of one of the studies highlighted that various models performed comparatively [70–72]. Finally, one study reported higher accuracy in post-treatment quality of life with the aid of an ML technology, but they noted that the 95% confidence intervals do overlap between the aided and unaided groups [66].

#### Neurodegenerative conditions

There were four records which reported on four studies looking into neurodegenerative conditions, including one protocol. Two studies looked at patients with Parkinson’s disease [73, 76]. One study looked at patients with multiple sclerosis and highlighted the utility of PROMs, but did not provide any measure of statistical certainty [74]. The one protocol was of a study looking at patients with spinal muscular atrophy [75].

Seven unique PROM tools were used in these studies, with the Parkinson’s Disease Questionnaire 39 being used twice and all other tools only once (Table 2). The Parkinson’s Disease Questionnaire 39 was the only PROM tool which was highlighted as an outcome measure of interest and a significant predictor of the studies’ outcomes (Table 2). There was no ML technique that was more popular than any other, but Long Short-Term Memory- recurrent NN was the only technique which was highlighted as best performing (Table 2). There were no special observations relating to these studies.

#### Mental health

There were seven records relating to seven mental health studies. Two studies looked at patients with depression in general [77, 79]. One highlighted PROMs as important baseline features in predicting both quality of life and costs associated with usual and blended therapy treatments [77]. The other looked at data from two trials of depression treatment [79]. One study looked at depressive symptoms in epilepsy patients [78]. One study looked at the risk factors of poor mental health outcomes in outpatients managed for COVID-19 [80]. One study looked at depressive symptoms in patients with opioid use disorders with the aim of predicting re-admission [81]. One study, looked at potential responders to a pharmacological agent studied for its application in the care of patients with alcohol use or major depressive disorders [83]. One study looked at psychological resilience in breast cancer patients to develop a clinical decision support tool [82]. It is a sibling study of one of the studies discussed in the cancer section [66].

**Fig. 7:**
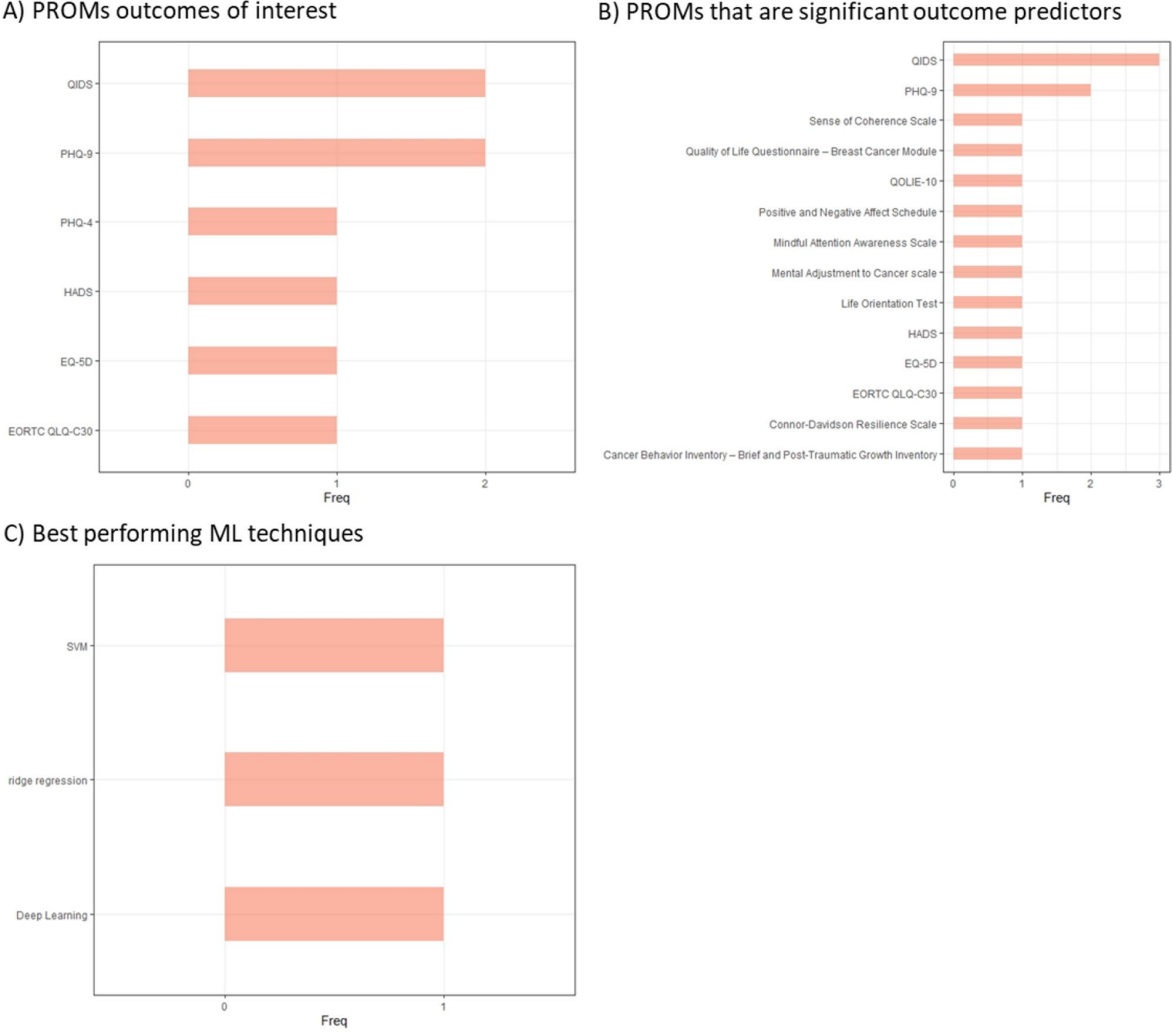
Key PROMs and ML techniques in studies of mental health patients. (**A**) PROMs that studies identified as outcomes of interest (**B**) PROMs which studies identified as significant outcome predictors (**C**) ML techniques that studies highlighted as best performing when more than one ML technique was investigated. European Organisation for the Research and Treatment of Cancer Quality of Life Questionnaire– Core Questionnaire (EORTC QLQ-C30), EuroQoL 5-Dimension (EQ-5D), Hospital Anxiety and Depression Scale (HADS), Patient Health Questionnaire (PHQ), Quick Inventory of Depressive Symptomatology (QIDS), Quality of Life in Epilepsy Inventory-10 (QOLIE-10), Support Vector Machine (SVM).

Across these studies 21 unique PROM tools were used, with Quick Inventory of Depressive Symptomatology and Patient Health Questionnaire-9 both used three times, and EQ-5D twice; all other tools were only used once (Table 2). Of the most frequently investigated ML methodologies, four studies investigated Random Forest methodology, three boosting, while K-Nearest Neighbor and Logistic Regression were both looked at twice (Table 2). Quick Inventory of Depressive Symptomatology and Patient Health Questionnaire-9 were the most investigated PROM outcome measures of interest and most frequently identified PROMs tools that were significant predictors of the studied outcomes (Fig. 7). No ML technique stood out as the most frequently best performing approach (Fig. 7). One study found that PROM scores were important predictors in some, but not all ML models predicting patient re-admission [81]. Another study noted patients with PROM scores indicating more depressive symptoms but better subjective quality of life responded best to treatment [78].

#### Other conditions

There were 11 records which related to 11 studies of a range of conditions that did not fit into any of the previously described categories, with two of these, being protocols. Two records pertained to studies looking at stroke patients undergoing such therapies as robot-assisted therapy and mirror therapy. One study described the range of explored therapies as ‘contemporary task-oriented’ [93]. The other looked at ‘sensorimotor rehabilitation interventions’ [91]. Two records described studies looking at the effect of different treatment regimens on patients suffering from rheumatoid arthritis. One study compared therapy with golimumab to therapy with infliximab, using data from a pragmatic trial to look at disease activity [85]. The second study looked at patient response to methotrexate treatment [87]. A single study looked at predicting breathlessness improvement using functional brain imaging [88]. One study explored the use of ML in the critical care setting and identified a range of risk factors for outcome after 6-weeks and 6-months [86]. One study looked at outcomes in patients suffering from post-COVID-19 syndrome [94]. A single study looked at predicting outcomes after bariatric surgery [84]. The authors did not provide any measure of statistical certainty, and as such it is not possible to comment how important any of these measures were as predictors. Finally, one study considered cytokine response and patient recovery after snake bites [90]. One protocol described a study on fall related emergencies in the care of the elderly [92]. The other protocol looked at a study predicting arrhythmic events and cardioverter-defibrillator therapy [89].

As this is a heterogonous collection of studies, information on the most frequently used PROM tools and ML approaches is not provided.

### Discussion

#### ML approaches to PROMs

One notable observation from the identified studies was that there was no clear ML approach which appeared to be more effective at predicting outcomes. Consider for example Kunze’s hip arthroscopy studies [22–25]. While they all evaluated data from patients with similar clinical indications and utilised similar PROM tools, a variety of ML approaches have been found to provide the best models in each study. Additionally, across the reported studies, researchers used a broad range of ML approaches, with studies often testing more than one approach and no one technique emerging as the preferred ML methodology across studies. These two observations suggest that researchers wishing to develop models for use in their own institutions should not solely rely on copying the approach which was reported to provide the best model in any previous study.

This review provides a summary of the ML techniques that have been previously used in combination with PROMs data. Researchers looking to apply ML techniques in their clinical settings can see from Table 2 which techniques proved to be most successful in the past in their clinical areas or with the PROMs that are currently collected at their institutions. This can help focus the research effort of those who only have the resources to investigate a limited range of ML techniques in their practice, but do not want to just copy a best-performing past approach. Nevertheless, it is likely that obtaining high quality reliable data is likely to be the biggest challenge when developing such ML models.

This review did not present any specific performance metrics for the models described in the identified studies. There are several key reasons for this. The primary aim of the review was to identify what has been done previously in relation to PROMs and ML, rather than to perform an evidence synthesis to assess the specific performance of the identified models. It is also not clear what performance threshold is good enough for a model, and as such models are best considered within the context of specific clinical contexts, rather simple summaries. For example, where an ML model might be used to help decide whether to give a patient treatment X or treatment Y, the degree of confidence we might wish to have in a model will depend on the risk and benefit profiles of both interventions. When considering two medications with similar risk profiles and where treatment can be easily changed from one to another, a clinician might be content to accept the advice of a worse performing model than when deciding whether or not to amputate a limb.

#### Pre-operative PROMs

A theme which emerged amongst the included records was that often one of the most influential predictors of a post-intervention PROM score was the pre-intervention PROM score; of the 37 studies that used at least one PROM tool as its outcome score (i.e. excluding protocols and studies that only utilised PROMs as predictors), 22 studies reported at least one post-intervention PROM score to have its pre-intervention counterpart as an important predictor. This suggests that the baseline wellbeing of a patient is the best predictor of their post-intervention wellbeing. Yet, there is a need to be careful not to conflate this with the impact of an intervention. For example, one study looking at lumbar spondylitis patients noted that the benefit of the intervention was greater in the subset of patients that were regarded as being in a worse health condition at the start of the study [39, 40]. The patients that might benefit most from the intervention might not be the same as those that will have the best PROM scores after it. Moreover, not all post-intervention PROM scores were best predicted by their pre-intervention counterparts. While this suggests that collection of pre-intervention PROM scores might be helpful when predicting patient outcomes, these scores will not always prove useful in such endeavours.

#### Study limitations

While this review utilised a comprehensive search of the literature, it is affected by a range of limitations. Abstracts often do not report all the variables assessed in a study. This means that studies might have been wrongly rejected during the abstract sift if the abstract omitted to indicate that PROMs had been utilised in a study. This might have particularly affected studies which utilised a range of variables, but did not report these in detail in the abstract. Considering the systematic search, a wide range of terms were used to identify relevant publications. However, some studies may have been missed where relevant concepts were described using alternative free text terms or controlled vocabulary. This might have been the case with the aforementioned review, though it is also possible that the outcomes reported by some of these publications did not align with our review scope [26]. Moreover, the review looked at the application of ML and PROMs to predicting post-intervention outcomes from pre-intervention data, where PROMs have been either a potential predictor and/or outcome measure. As such it excluded studies which looked at the diagnostic ability of ML applications utilising PROMs data, or predicting long-term outcomes from short-term post-intervention data. Due to the extensive nature of identified studies, the review did not report on other predictor or outcome factors. The review did also not report specific model performance, as this information is of little relevance without appreciating the broader clinical context of each ML model’s use. Finally, to focus the review, we did not look at studies assessing care-giver wellbeing or where the outcome of interest was financial well-being.

Further research, through larger scale studies or meta-analysis might help to identify best performing ML techniques as well as PROMs that are most suitable for use in ML models. Nevertheless, the choice of PROMs tools might be dictated by other factors, such as their use in clinical practice or historical adoption reasons, and consequently the fact that studies might have reported success in using specific PROMs with ML techniques might not represent a strong enough incentive for adoption in clinical practice. Similarly, the choice of which ML techniques to use might also relate to whether one is interested in a regression problem or classification problem, and what data one has available to be used in a potential model. Consequently, such information can provide useful pointers to researchers, clinicians and healthcare decision-makers, but is unlikely to replace local evaluation of various models. Finally, it is important to remember that new PROMs and ML techniques might be developed, and such tools will need to be evaluated and considered in future research.

## Conclusions

This review summarised 82 records describing 73 studies that predicted patient post-intervention outcomes using a combination of ML techniques and PROM tools, where PROMs were either used as a predictor or considered as an outcome. The biggest group of identified studies related to orthopaedics, particularly to hip and knee surgery. Even when authors studied patients with similar conditions, they often employed a range of PROM tools and ML techniques. The variety of approaches used and results of these studies, suggest that while it might be possible to develop clinically useful models, there is no one best ML technique. Those wishing to implement ML-based decision support tools should evaluate their data using a wide range of approaches to see which perform best, rather than simply replicating a published model.

## Electronic supplementary material

Below is the link to the electronic supplementary material.

Supplementary Material 1

## Data Availability

No datasets were generated or analysed during the current study.

## Abbreviations

Adaboost: Adaptive Boosting
AI: Artificial Intelligence
ASES: American Shoulder and Elbow Surgeons score
AUDIT: Alcohol Use Disorders Identification Test
COMI: Core Outcome Measures lndex
CTCAE: Common Terminology Criteria for Adverse Events
DASH: Disabilities of Arm, Shoulder and Hand score
DT: Decision Trees
ENPLR: Elastic-Net Penalised Logistic Regression
ENR: Elastic Net Regression
EORTC QLQ-C30: European Organisation for the Research and Treatment of Cancer Quality of Life Questionnaire: Core Questionnaire
EQ-5D: EuroQoL 5-Dimension
FABQ: Fear Avoidance Belief Questionnaire
FACS-D: Fear-Avoidance Components Scale
GAD: Generalised Anxiety Disorder
GBoost: Gradient Boosting
GLMM: Generalised Linear Mixed Model
GNB: Gaussian Naïve Bayes
HADS: Hospital Anxiety and Depression Scale
HAQ: Health Assessment Questionnaire
HOS: Hip Outcome Score
IEQ: Injustice Experience Questionnaire
iHOT: Hip Outcome Tool
IKDC: International Knee Documentation Committee subjective form
JOABPEQ: Japanese Orthopedic Association Back Pain Evaluation Questionnaire
KNN: K-Nearest Neighbor
KOOS: Knee Injury and Osteoarthritis Outcome Score
KOS-ADL: Knee Outcome Survey–Activities of Daily Living
LR: Logistic Regression
MDASI: MD Anderson Symptom Inventory
ML: Machine Learning
mHHS: Modified Harris Hip Score
MHQ: Michigan Hand outcomes Questionnaire
mJOA: modified Japanese Orthopedic Association scale
MSPQ: Modified Somatic Perception Questionnaire
MuARS: Multivariate Adaptive Regression Splines
NDI: Neck Disability Index
NN: Neural Networks
ODI: Oswestry Disability Index
OHS: Oxford Hip Score
OKS: Oxford Knee Score
OSS: Oxford Shoulder Score
PCS: Pain Catastrophizing Scale
PHQ: Patient Health Questionnaire
PDQ-39: Parkinson’s Disease Questionnaire 39
PROMIS: Patient-Reported Outcomes Measurement Information System
PSEQ: Pain Self Efficacy Questionnaire
PROMs: Patient Reported Outcome Measures
QDA: Quadratic Discriminant Analysis
QIDS: Quick Inventory of Depressive Symptomatology
QOLIE-10: Quality of Life in Epilepsy Inventory-10
RF: Random Forest
RMDQ: Roland Morris Disability Questionnaire
SES: Self Efficacy Scale
SF: Short Form
SGD: Stochastic Gradient Descent
SIS: Stroke Impact Scale
SPADI: Shoulder Pain and Disability Index
SVM: Support Vector Machine
TSK-17: Tampa Scale for Kinesiophobia
UCLA: University of California at Los Angeles shoulder score
VR-12: Veterans RAND 12-item Health Survey
WHODAS: WHO Disability Assessment Schedule
WOMANC: Western Ontario and McMaster University Osteoarthritis Index
WORC: Western Ontario Rotator Cuff index
XGBoost: Extreme Gradient Boosting
