# Supplementary material for: A narrative review of the use of PROMs and machine learning to impact value-based clinical decision-making
Source: BMC Med Inform Decis Mak. 2025 Jul 4;25:250. doi: 10.1186/s12911-025-03083-8 (PMC12226851; doi:10.1186/s12911-025-03083-8)
Supplement: Supplementary file 1 — Supplementary Material 1 [file 12911_2025_3083_MOESM1_ESM.docx]

# Appendix 1

Ovid MEDLINE(R) ALL <1946 to October 10, 2023>

| **#** | **Query** | **Results from 11 Oct 2023** |
| --- | --- | --- |
| 1 | exp Artificial Intelligence/ | 180,583 |
| 2 | exp Random Forest/ | 354 |
| 3 | exp Neural Networks, Computer/ | 61,328 |
| 4 | (Artificial* Intelligen* or AI or machine learning or deep learning or neural network* or convolutional network*).tw. | 243,581 |
| 5 | 1 or 2 or 3 or 4 | 326,553 |
| 6 | "Outcome Assessment (Health Care)"/ | 82,418 |
| 7 | "Quality of Life"/ | 273,936 |
| 8 | (PROM or PROMs or "quality of life" or "health?related quality" or "health related quality" or QOL or HRQL or HRQOL).tw. | 388,249 |
| 9 | (patient* adj2 report* adj (outcome* or measure*)).tw. | 35,190 |
| 10 | 6 or 7 or 8 or 9 | 545,457 |
| 11 | (decision support and (tool* or technique*)).tw. | 8,836 |
| 12 | Decision Support Techniques/ | 22,526 |
| 13 | Predictive Value of Tests/ | 223,504 |
| 14 | ((predict* or identif*) adj2 clinic*).tw. | 101,839 |
| 15 | 11 or 12 or 13 or 14 | 344,523 |
| 16 | 5 and 10 and 15 | 266 |
| 17 | exp animals/ not humans.sh. | 5,161,760 |
| 18 | 16 not 17 | 265 |
| 19 | limit 18 to (english language and yr="2000 -Current") | 244 |

Embase <1974 to 2023 October 10>

| **#** | **Query** | **Results from 11 Oct 2023** |
| --- | --- | --- |
| 1 | artificial intelligence/ | 64,017 |
| 2 | artificial intelligence software/ | 424 |
| 3 | random forest/ | 25,084 |
| 4 | convolutional neural network/ | 26,281 |
| 5 | artificial neural network/ | 52,276 |
| 6 | (Artificial* Intelligen* or AI or machine learning or deep learning or neural network* or convolutional network*).tw. | 293,342 |
| 7 | 1 or 2 or 3 or 4 or 5 or 6 | 336,424 |
| 8 | outcome assessment/ | 871,537 |
| 9 | "quality of life"/ or "quality of life assessment"/ | 635,463 |
| 10 | (PROM or PROMs or "quality of life" or "health?related quality" or "health related quality" or QOL or HRQL or HRQOL).tw. | 615,178 |
| 11 | (patient* adj2 report* adj (outcome* or measure*)).tw. | 59,381 |
| 12 | 8 or 9 or 10 or 11 | 1,611,521 |
| 13 | (decision support and (tool* or technique*)).tw. | 11,884 |
| 14 | decision support system/ | 27,457 |
| 15 | "prediction and forecasting"/ | 18,774 |
| 16 | ((predict* or identif*) adj2 clinic*).tw. | 153,722 |
| 17 | 13 or 14 or 15 or 16 | 204,024 |
| 18 | 7 and 12 and 17 | 1,238 |
| 19 | limit 18 to (english language and yr="2000 -Current") | 1,228 |

Cochrane Library

Date Run: 11/10/2023 14:24:12

ID Search Hits

#1 MeSH descriptor: [Artificial Intelligence] 1 tree(s) exploded 2942

#2 MeSH descriptor: [Random Forest] 1 tree(s) exploded 54

#3 MeSH descriptor: [Neural Networks, Computer] 1 tree(s) exploded 537

#4 (Artificial* Intelligen* or AI or machine learning or deep learning or neural network* or convolutional network*):ti,ab,kw 12115

#5 #1 or #2 or #3 or #4 13458

#6 MeSH descriptor: [Outcome Assessment, Health Care] 2 tree(s) exploded 192216

#7 MeSH descriptor: [Quality of Life] 1 tree(s) exploded 44212

#8 (PROM or PROMs or "quality of life" or "health-related quality" or "healthrelated quality" or "health related quality" or QOL or HRQL or HRQOL):ti,ab,kw 154681

#9 (patient* NEAR/2 report* NEXT (outcome* or measure*)):ti,ab,kw 14463

#10 #6 or #7 or #8 or #9 329931

#11 (decision support and (tool* or technique*)):ti,ab,kw 3293

#12 MeSH descriptor: [Decision Support Techniques] 1 tree(s) exploded 3988

#13 MeSH descriptor: [Predictive Value of Tests] 1 tree(s) exploded 8768

#14 ((predict* or identif*) NEAR/2 clinic*):ti,ab,kw 24399

#15 #11 or #12 or #13 or #14 38225

#16 #5 and #10 and #15 with Cochrane Library publication date from Jan 2000 to present 168

Scopus

(( ABS ( ( "artificial* intelligen*" ) OR ( ai ) OR ( "machine learning" ) OR ( "deep learning" ) OR ( "neural network*" ) OR ( "convolutional network* " ) OR ( "random forest* " ) ) AND ABS ( ( "outcome assessment" ) OR ( "patient reported outcome" ) OR prom OR proms OR ( "quality of life" ) OR ( "health-related quality" ) OR ( "healthrelated quality" ) OR ( "health related quality" ) OR qol OR hrql OR hrqol ) AND ABS ( ( "decision support" ) OR ( "predictive value" ) OR ( "predict clinical" ) OR ( "identify clinical" ) ) )) OR (( TITLE ( ( "artificial* intelligen*" ) OR ( ai ) OR ( "machine learning" ) OR ( "deep learning" ) OR ( "neural network*" ) OR ( "convolutional network " ) OR ( "random forest " ) ) AND TITLE ( ( "outcome assessment" ) OR ( "patient reported outcome" ) OR prom OR proms OR ( "quality of life") OR ( "health-related quality" ) OR ( "healthrelated quality" ) OR ( "health related quality" ) OR qol OR hrql OR hrqol ) AND TITLE ( ( "decision support" ) OR ( "predictive value" ) OR ( "predict clinical" ) OR ( "identify clinical" ) ) )) AND PUBYEAR > 1999 AND PUBYEAR < 2025 AND ( LIMIT-TO ( LANGUAGE,"English" ) )

IEEE Xplore

(( "artificial* intelligen*" ) OR ( ai ) OR ( "machine learning" ) OR ( "ML" ) OR ( "deep learning" ) OR ( "neural network*" ) OR ( "convolutional network* " ) OR ( "random forest" ) ) AND (( "outcome assessment" ) OR ( "patient reported outcome" ) OR prom OR proms OR ( "quality of life") OR ( "health*related quality" ) OR ( "health related quality" ) OR qol OR hrql OR hrqol) AND ( ( "decision support" ) OR ( "predictive value" ) OR ( "predict clinical") OR ( "identify clinical" ) )

Note: search was not restricted to 2000, as oldest item was from 2005

ACM Digital Library

[[Abstract: "artificial intelligence"] OR [Abstract: ai] OR [Abstract: "machine learning"] OR [Abstract: "ml"] OR [Abstract: "deep learning"] OR [Abstract: "neural network"] OR [Abstract: "convolutional network"] OR [Abstract: "random forest"]] AND [[Abstract: "outcome assessment"] OR [Abstract: "patient reported outcome"] OR [Abstract: prom] OR [Abstract: proms] OR [Abstract: "quality of life"] OR [Abstract: "health-related quality"] OR [Abstract: "healthrelated quality"] OR [Abstract: "health related quality"] OR [Abstract: qol] OR [Abstract: hrql] OR [Abstract: hrqol]] AND [[Abstract: "decision support"] OR [Abstract: "predictive value"] OR [Abstract: predict* clinical*] OR [Abstract: identif* clinical*]] AND [E-Publication Date: (01/01/2000 TO *)]

*Note: Searched The ACM Guide to Computing Literature (3,579,556 records)*
